# Effect of cognitive behavioral social skills training on functioning in schizophrenia: protocol for an individual participant data meta-analysis of randomized-control trials

**DOI:** 10.1186/s13643-026-03124-x

**Published:** 2026-03-05

**Authors:** Matthias Pillny, Jason Holden, Dan Devoe, Peter Link, Eric Granholm

**Affiliations:** 1 Clinical Psychology and Psychotherapy, Institute of Psychology, Faculty of Psychology and Human Movement Science, Universität Hamburg, Hamburg, 20146 Germany; 2 Department of Psychology, Institute of Psychiatry, Psychology & Neuroscience, King’s College London, London, United Kingdom; 3 Department of Psychiatry, University of California, San Diego, La Jolla, CA USA; 4 Department of Psychology, Mount Royal University, Calgary, Canada

**Keywords:** Schizophrenia, CBSST, Functioning, Social recovery, Negative symptoms, Evidence based, Psychotherapy, Psychosis, Amotivation, Defeatist beliefs, Social skills

## Abstract

**Background:**

Cognitive Behavioral Social Skills Training (CBSST) is a targeted psychological intervention designed to improve daily functioning and to address negative symptoms in individuals diagnosed with schizophrenia. Despite evidence from clinical trials suggesting beneficial effects of CBSST on functioning and negative symptoms, the overall efficacy of CBSST remains to be quantified. Furthermore, potential moderators and mediators of treatment outcomes remain elusive. This protocol outlines an individual participant data meta-analysis (IPD-MA) with the objective to examine the efficacy of CBSST on psychosocial functioning in schizophrenia.

**Method:**

In accordance with the Preferred Reporting Items for a Systematic review and Meta-Analysis of Individual Participant Data (PRISMA-IPD) guidelines, we will conduct a systematic literature search and employ two-stage and one-stage meta-analytical approaches. The meta-analytical models will evaluate the overall effect of CBSST relative to control treatments in randomized-control trials, identify participant-level (e.g., age, cognitive impairment) and study-level (e.g., individual vs. group settings) predictors of change, and explore the mechanisms that mediate improvement in functioning, such as skills acquisition and cognitive restructuring of defeatist attitudes. Furthermore, the analysis will attempt to determine the optimal amount of CBSST sessions required to enhance functioning and evaluate the impact of patient-level factors driving delivered dosage.

**Discussion:**

The objective of this study is to contribute to the existing literature by addressing the current gaps in understanding the efficacy of CBSST and identifying critical factors for treatment success. Our findings will have the potential to inform personalized treatment planning and the development of clinical guideline recommendations focusing on functional outcomes and negative symptoms in people with schizophrenia.

**Systematic review registration:**

PROSPERO CRD42024605353.

Schizophrenia is a complex and debilitating mental disorder characterized by a range of cognitive, emotional, and social dysfunctions that are associated with the decline in an individual’s daily-life functioning. The negative symptoms of schizophrenia (i.e., avolition, anhedonia, asociatlity, blunted affect, and alogia; [1]) account for much of the variance in poor functioning outcomes (e.g., [2]) and are a largely unmet treatment need. Antipsychotic medication is suspected to impede long-term functioning [3], while the evidence base for psychological interventions is modest with small and short-term effect sizes on negative symptoms [4]. Consequently, despite advances in psychological treatment of delusions and hallucinations [5, 6], at least 50% of individuals with schizophrenia continue to experience pronounced negative symptoms and consequently significant impairments in everyday skills that are required for independent living, such as their ability to maintain relationships, engage in employment, and navigate everyday challenges [7–9].

Cognitive Behavioral Social Skills Training (CBSST, [10]) has emerged as a promising evidence-based intervention. It combines cognitive-behavioral techniques with social skills training to address the cognitive and social deficits that are common in schizophrenia and have been shown to be associated with negative symptoms and functional impairment [11–18]. CBSST therefore emphasizes the importance of skill acquisition and incorporates elements of cognitive restructuring to challenge dysfunctional thinking patterns that impede social engagement (e.g., [19, 20]). Thus, by fostering social interaction and general problem-solving abilities and addressing the cognitive barriers to effective communication and pursuit of recovery goals, CBSST offers a targeted approach to improving psychosocial functioning in individuals with schizophrenia-spectrum disorders.

Prior randomized-control trials (RCTs) have demonstrated that compared to treatment as usual or goal-focused supportive contact, CBSST is effective at improving functioning and, in clinical trials with non-geriatric samples, was effective at improving amotivation, asociality, and anhedonia domains of negative symptoms in people with schizophrenia at posttreatment and up to 1-year follow-up [21–25]. Moreover, studies investigating mechanisms of change in CBSST have found that significant reductions in defeatist performance beliefs mediated improvement in experiential negative symptoms and functioning, and participants with more severe defeatist beliefs prior to treatment showed better outcomes in CBSST [21, 22, 26]. While these findings suggest that—at the study level—CBSST is effective in improving psychosocial functioning and in alleviating experiential negative symptoms and defeatist beliefs, a comprehensive meta-analytic aggregation of the available evidence is currently lacking.

Previous meta-analyses investigating the effects of psychological interventions in general on functioning outcomes in patients with schizophrenia have included only a subset of the available evidence on CBSST, which might have led to an incomplete understanding of the true efficacy of CBSST (e.g., [27]). Furthermore, previous evidence indicates that CBSST may have varying effects on specific populations, including middle-aged or older patients [21, 23] and those with particularly pronounced deficits in executive function [28] and more severe defeatist attitudes and negative symptoms [24, 29]. For instance, male patients have more prevalent negative symptoms and social functional impairment compared to other genders [30–32]. Thus, a more nuanced understanding of the efficacy of CBSST could be achieved by investigating potential participant-level moderators of treatment effects across different studies. This could facilitate an improved understanding of the underlying mechanisms of change and contribute to the development of differential indications and personalized treatment approaches. However, classical meta-analyses are unable to account for the complexity and heterogeneity of CBSST’s effects at the participant level. This is due to the fact that these focus on synthesizing overall treatment effects across studies by aggregating average effect sizes at the study level. This “study-level approach” does not account for variation in participant-level characteristics and may obscure interactions between participant characteristics and treatment outcomes.

An individual participant data (IPD) meta-analysis allows us to analyze raw data collected from individual studies and enables the examination of participant-level predictors that traditional aggregate meta-analyses do not readily account for [33]. Moreover, this approach enhances statistical power by leveraging larger, pooled data sets, allowing for more precise analyses of treatment effects based on varying patient-level characteristics and can yield more reliable and robust conclusions [34]. Thus, a comprehensive IPD meta-analysis of CBSST will bridge the gap of knowledge on the overall efficacy of CBSST on functioning as well as on study- and participant-level predictors of change.

The aim of this study is to synthesize existing evidence through an individual participant data meta-analysis (IPD-MA) by (1) evaluating the overall efficacy of CBSST on social functioning outcomes in people with schizophrenia; (2) identifying patient-level (e.g., age, gender, cognitive functioning, defeatist attitudes) and study-level (e.g., blended digital intervention, group vs. individual format, active vs. passive comparator) predictors of treatment efficacy; (3) exploring the mechanisms of change, such as level of skill learning and change in cognitive attitudes, in facilitating improvements in functioning; and (4) determining the dose of CBSST (number of sessions) needed to improve functioning and whether dosage is impacted by patient-level predictors (e.g., age, cognitive impairment). Our main hypothesis is that CBSST will be superior to control condition in improving functioning and secondary outcomes.

## Method

Methods for this IPD-MA are based on the Preferred Reporting Items for a Systematic review and Meta-Analysis of Individual Participant Data [35]. The protocol has been submitted to PROSPERO on October 23, 2024 (*CRD42024605353*). We will update the record with any changes that will be made to the original protocol.

## Eligibility criteria

### Participants

We will include participants (as defined in the original studies) with the diagnosis of a schizophrenia-spectrum disorder (i.e., schizophrenia, schizoaffective disorder, schizophreniform disorder) according to ICD-9, ICD-10, DSM IV, or DSM 5. All kinds of diagnostic procedures and all kinds of comorbid disorders will be accepted. Populations with subthreshold psychotic symptoms (e.g., clinical high-risk populations or attenuated psychosis syndrome) will be excluded. There will be no restrictions regarding demographics (e.g., age, gender, race).

### Intervention

CBSST is an evidence-based psychosocial treatment designed for individuals with schizophrenia and other schizophrenia-spectrum disorders [10]. It integrates cognitive behavioral therapy techniques with social skills training to address both the cognitive and social functioning deficits common in schizophrenia. CBSST is typically delivered in group sessions, allowing for peer support, observation of social interactions, and skill practice in a safe environment. Groups usually meet weekly over several months, but other formats such as individual sessions delivered on Assertive Community Treatment teams [36] have also been tested. Moreover, other trials have combined forms of CBSST with nasal administration of oxytocin [37], compensatory cognitive training [24, 29], or mobile digital interventions [38, 39]. In the original manual, three modules focus on specific skill sets, including recognizing and changing dysfunctional thoughts and beliefs, improving social communication skills, and solving everyday problems related to recovery goal achievement. The techniques involve a mix of psychoeducation, skills training, and practical exercises. Specifically, the three CBSST modules are as follows:

1. *The cognitive skills module* encourages participants to recognize and challenge defeatist thoughts that can impede the motivation to engage in social situations or work on recovery goals. It is foundational, as the techniques taught are integrated throughout the other two modules. Core principles include psychoeducation about the generic cognitive model (i.e., the link between thoughts, feelings, and behavior), about automatic thoughts, and common mistakes in thinking such as overly pessimistic expectations (e.g., “I won’t like it”) and defeatist beliefs (e.g., “I can’t succeed”). Techniques that are trained to challenge these thoughts include behavioral experiments and mnemonic aids (e.g., “the 3Cs: Catch it, Check it, Change it” for challenging thoughts).
2. *The social skills module* aims to improve social interaction and communication skills. Participants are encouraged and guided to practice specific behaviors and techniques needed for successful social functioning in a variety of settings, such as talking to others, handling conflicts, and making requests. The key techniques include role-play exercises, behavioral rehearsal of social skills such as active listening and assertive communication, and feedback reinforcement.
3. *The problem-solving skills module* aims to improve the participants’ skills in developing approaches to solving everyday problems. The goal is to equip individuals with the skills to effectively navigate challenges in their personal, social, and work lives. The mnemonic SCALE acronym (*S*pecify the problem, *C*onsider all possible solutions, *A*ssess the best solution, *L*ay out a plan, and *E*xecute and evaluate the outcome) is used to teach the standard five steps of problem-solving.

These modules are usually delivered sequentially but are integrated throughout the training to build a comprehensive skill set for managing daily life challenges and working on recovery goals. Setting a living, learning, working, or socializing recovery goal and breaking this long-term recovery goal down into short-term goals and goal steps that can be accomplished each week by using the skills in the three modules are the cornerstone of the CBSST intervention.

### Comparator

Any kind of comparator will be included. On the study level, comparators will be classified as either passive (i.e., treatment as usual/waitlist) or active such another psychosocial intervention or supportive counseling. The comparator classification will be coded in the data and tested as a covariate of the synthesized effect of CBSST on outcomes.

### Study type

We will include RCTs that compared CBSST to a control condition, as defined above, in patients diagnosed with a schizophrenia-spectrum disorder. Quasi-RCTs, case studies, and crossover trials will be excluded. There will be no restrictions to study context or setting. Information about context and setting will be coded in the data and tested as covariates in moderation analyses. These will include (a) in- vs. outpatient settings, (b) individual vs. group therapy, (c) research clinic vs. other services, and (d) “classical” CBSST vs. augmented CBSST (e.g., by compensatory cognitive training or mobile digital interventions).

### Outcomes

The outcomes were selected and defined in consideration of the main treatment targets proposed in the CBSST manual [10] and after pilot screening of study articles.

### Primary outcomes

The primary outcome will be the level of functioning assessed at posttreatment, 6-month, and 12-month follow-up, with the 12-month assessment defined as the primary endpoint. Eligible functioning measures are validated scales that capture a type of psychosocial functioning (i.e., an individual’s ability to perform and manage daily activities, maintain social relationships, and fulfill social roles within their environment). This encompasses a wide range of skills and behaviors that allow a person to effectively interact with others, manage emotions, and cope with the demands of daily life. Measures of global functioning, role, or social functioning will be eligible.

### Secondary outcomes

We will also collect data on the following secondary outcomes, if available:

1. Severity of negative symptoms
  a According to the two-factor model (i.e., experiential and expressive negative symptoms; [40])
  b According to the five-factor model (i.e., avolition, anhedonia, asociality, blunted affect, and alogia; [1])
2. Severity of positive symptoms
3. Severity of depressive symptoms
4. Severity of defeatist performance beliefs
5. Severity of asocial beliefs
6. Cognitive functioning (global and attention/processing speed, verbal learning and memory, and executive function domains)
7. CBSST skill learning
8. Social communication skills
9. Quality of life

### Information sources and search strategy

A systematic literature search following Preferred Reporting Items for a Systematic review and Meta-Analysis of Individual Participant Data (PRISMA-IPD; [35]) will be conducted. To identify relevant records, three databases will be searched. PubMed will be searched via MEDLINE using the “.mp” field extension, which includes nine search fields (Title, Abstract, Heading Word, Table of Contents, Key Concepts, Original Title, Tests & Measures, MeSH). PsycINFO will be searched via NCBI using the “All Fields” preset. Embase will be searched using the “ALL” field command. Additionally, clinical trial registries such as ClinicalTrials.gov will be searched for unpublished data. For all databases, the search will be limited to records dating back to 2005, written in either English or German. The query will include a term referring to psychotic disorders and a term referring to CBSST to appear concurrently in the search fields. Manual reference section citation searches of included articles and previous review articles will be conducted. Google Scholar will be used for additional manual searches. A medical librarian will be consulted with the protocol at Universität Hamburg before searches are conducted. Identified records will be handled using AI-assisted tools such as Covidence or Rayan to remove duplicates and to perform the title and abstract screening.

We will contact the authors of eligible studies and request anonymized IPD and any additional relevant studies or information. E-mails will be sent to the first and/or corresponding author of the studies. In the event of nonresponse, we will send reminders and attempt to contact other authors or use alternative communication methods, such as telephone. Should there be persistent uncertainty regarding the eligibility criteria and/or IPD availability due to inadequate author responses despite our efforts, the study will be excluded from the analysis. In cases where IPD remain unavailable, we will incorporate available aggregate-level data within the two-stage approach (see the “Data synthesis” section). Missingness in the one-stage analyses will be appropriately addressed and reflected in the assessment of confidence in the evidence (see the “ Confidence in the evidence” section).

## Study selection and data collection

### Study selection

M. P. and D. D. will independently screen the records for inclusion and apply the eligibility criteria following a two-step screening process. First, titles and abstracts will be screened to identify potentially eligible studies. Second, full texts of potentially relevant or unclear records will be obtained and assessed against the eligibility criteria. Disagreements between the individual judgments will be resolved by discussion with E. G. and J. H. When required, further information will be requested from study authors. Decisions will be recorded in a table documenting the study title, the study authors, the year of publication, and the reason for exclusion. The process of study selection will be reported with a PRISMA-IPD flow diagram.

### Data collection and extraction

M. P. and E. G. will request IPD from all selected records. P. L. will merge harmonized individual participant data of all included records and add a categorical trial identifier assigning each participant to the respective trial. Data will be merged into a long format file with rows by participant and study visit/time points. An additional categorical variable will be added identifying the number of the respective study visit. Furthermore, another continuous variable will contain information about the follow-up duration from baseline in months. The final dataset will be validated by M. P. and J. H. by examining it for missing values, outliers, and duplicated values, and the adequacy of randomization (if possible with the available data), and cross-checking with the summary statistics reported in the published studies. In case of any discrepancies, we will collaborate with the study authors to address and resolve the issues. When IPD is not available, two independent reviewers will extract aggregated data from the original reports. Please see preregistration record for the full list of extracted variables.

### Risk‐of‐bias assessment

Risk of bias in individual studies will be assessed by M. P. and D. D. using Version 2 of the Cochrane risk-of-bias tool for randomized trials (RoB 2; [41]). Any disagreement will be resolved by discussion with J. H. and E. G. RoB 2 uses signaling questions and an algorithm to help make judgements of “low risk,” “some concerns,” or ‘high risk” related to five domains:

1. Bias arising from the randomization process
2. Bias due to deviations from intended interventions
3. Bias due to missing outcome data
4. Bias in measurement of the outcome
5. Bias in selection of the reported result

An overall risk of bias will be determined by the tool’s algorithm. These judgements will be used to inform the assessment of within-study bias in the evaluation of the confidence of the evidence (see “ Confidence in the evidence”) and will be included in between-study moderator analyses to test whether the effect of CBSST is dependent on a study’s risk of bias.

### Assessment of applicability

According to our eligibility criteria, the included RCTs are expected to provide evidence regarding both the efficacy in standardized research contexts and the effectiveness of interventions in real-world settings. To further evaluate potential issues related to applicability, two independent reviewers will employ the Rating of Included Trials on the Efficacy-Effectiveness Spectrum (RITES) tool [42]. The RITES tool assesses domains such as participant characteristics, trial setting, intervention flexibility, and clinical relevance. Each domain is rated on a 5-point Likert scale, ranging from 1 (“strong emphasis on efficacy”) to 5 (“strong emphasis on effectiveness”). These ratings will inform the assessment of potential indirectness in the evidence (see the “ Confidence in the evidence” section).

### Confidence in the evidence

We will evaluate the certainty of the evidence using the GRADE (Grading of Recommendations, Assessment, Development, and Evaluations; [43]) approach for our main outcome, namely levels of functioning. The GRADE framework provides a systematic method for assessing the quality of evidence and the strength of recommendations. It considers factors such as risk of bias, inconsistency of results, indirectness of evidence, imprecision, and publication bias. Evidence is rated across four levels: high, moderate, low, and very low certainty. This approach ensures a transparent and structured process for evaluating how much confidence can be placed in the findings of the included studies, ultimately guiding the formulation of reliable conclusions and recommendations.

## Data synthesis

This IPD meta-analysis will include both a two- and a one-stage approach. The rationale for our statistical approach follows the recommendations for the practice of individual participant data meta-analyses [44] and the PRISMA-IPD criteria [35].

Continuous variables will be z-standardized within each trial prior to pooling. Categorical variables will be harmonized by aligning reference categories across trials to ensure consistent interpretation of regression coefficients.

Missing outcome data at posttreatment and follow-up assessments will be addressed using multiple imputation under the missing-at-random assumption. A total of 100 imputed datasets will be generated based on baseline functioning scores, age, sex, and treatment group. Each imputed dataset will include both observed and imputed standardized functioning scores for missing values. The imputed datasets will be analyzed separately using the prespecified model, and the resulting estimates will be combined according to Rubin’s rules [45]. Sensitivity analyses based on complete cases will be conducted to evaluate the robustness of the results and to examine potential differences between participants who complete posttreatment assessments and those who dropped out.

### Effect sizes

For the two-stage approach, means and standard deviations of outcomes will be transformed into standardized mean differences (SMD) [46] and corrected for overestimation using Hedge’s g [47]. Positive SMDs will indicate better outcomes in participants receiving CBSST, while negative SMDs reflect outcomes favoring control conditions. Effect sizes will be presented along with 95% confidence intervals. Number needed to treat will be estimated as a secondary measure of effect for functioning outcomes.

For the one-stage approach, the standardized regression-based beta coefficient of the treatment effect with its corresponding standard error and 95% confidence intervals will be reported as a measure of effect.

### Synthesis approach

#### The two-stage approach

First, we will synthesize a summary effect size of CBSST on primary functioning outcomes at the final follow-up of each study following the two-stage approach. In the first stage, IPD within a trial will be analyzed to generate a study-level summary effect (i.e., Hedge’s g; see the next section). In the second stage, the effect sizes from each trial will be aggregated across trials using conventional two-level random-effects meta-analytical methods, which accounts for between-study heterogeneity [47]. Heterogeneity of effect sizes will be evaluated using the Q-statistic and *I*^2^ [46, 48]. All statistical analyses within the two-stage approach will be conducted using the “metafor” package [49] implemented in RStudio.

#### The one-stage approach

In a second step, we will follow the one-stage approach by combining all individual participant data in a single meta-analysis based on a mixed linear regression model stratified by trial. These models are particularly suitable for investigating how treatment effects vary between individuals or groups and have improved ability to detect differences between groups of participants over two-stage meta-analyses due to higher statistical power. Furthermore, the one-stage approach allows to separate group-level and individual-level variance. For this IPD meta-analysis, we will estimate mixed linear models to predict functioning and secondary outcomes across the follow-up visits of each study. The model will include group (treatment vs. control group), study visit, and the interaction of both as fixed effects. Model specification will follow the maximal-random approach for multilevel modeling [50]. This ensures appropriate control of type 1 error rates and to allow for generalization of fixed effects across participants and trials. Because this approach models all theoretically justified sources of random variation, it provides more reliable and generalizable inferences about treatment effects. If model convergence can be achieved, the random-effects structure will include a random intercept for trial and participant, as well as a random slope for time (study visit). Model convergence will be evaluated based on the absence of optimization errors, stable variance–covariance estimates, and parameter estimates within reasonable bounds (i.e., variance components within finite, nonzero ranges and correlation parameters between −1 and 1 without boundary estimates). In cases of non-convergence, the random-effects structure will be simplified in a predefined sequence. First by removing the random slope for time, then by removing the random intercept for trial, and, finally, if necessary, by estimating a participant-level random intercept only. Each simplification step will be documented, and the final model specification used for each analysis will be reported in the supplementary materials along with model diagnostics. To evaluate the robustness of the findings, sensitivity analyses will be conducted by comparing estimates across alternative model specifications (i.e., maximal versus simplified random-effect structures). Results will be interpreted primarily based on the model with the best fit (i.e., *χ*^2^, AIC, BIC, marginal and conditional *R*^2^). These models will be estimated using the “lme4” and the “lmertest” packages implemented in RStudio [51, 52]. Please see Wilkinson notation below:

$$Functioning \sim group+study visit+group\times study visit+\left(study visit|participant\right)+\left(1|trial\right)$$

### Subgroup and meta-regression analyses

We will conduct a set of study-level subgroup and meta-regression analyses within the two-stage approach. Additionally, in the one-stage approach, we will test a set of participant-level covariates using meta-regressions by pooling data from all studies and directly examining the influence of individual participant characteristics. This dual approach allows for a thorough exploration of both study- and participant-level factors. Please see below for a list of subgroup and meta-regression tests.

#### Study level (two-stage approach)

Type of comparator, study setting, type of trial (efficacy vs. effectiveness), level of therapist training, and additional treatment modules (e.g., digital or cognitive remediation) will be tested by estimating separate meta-regression models at the second stage of the two-stage analysis.

#### Participant level (one-stage approach)

Diagnoses, age, gender, years of education, ethnicity/race, medication status (medicated/unmedicated), type of medication at baseline, chlorpromazine equivalent doses at baseline, depressive symptoms at baseline, positive symptoms at baseline, negative symptoms at baseline, illness status (first-episode, chronic), cognitive functioning at baseline, defeatist and asocial beliefs at baseline, psychosocial functioning at baseline, number of sessions received, therapist fidelity, and social skills at baseline will be tested as covariates and their interaction with the group by study visit effect in the one-stage models separately for each outcome and covariate. An additional exploratory model including all covariates will be estimated to determine the most relevant predictors of change.

### Mediation analyses

Defeatist performance beliefs, motivational negative symptoms, and skills acquisition will be tested as potential mediators of the improvement in functioning. Mediation analyses will be conducted using a one-stage multilevel structural equation modeling (SEM) approach using the lavaan package in R [53]. The SEM framework will be structured with individuals at level 1 nested within trials at level 2. Trials will be treated as clusters with between-trial variability modeled by random intercepts at level 2. To establish temporal ordering, mediators at posttreatment will be modeled as predictors of subsequent changes in functioning at either 6- or 12-month follow-ups, such that mediator assessments temporally precede outcome assessments. Only studies with appropriate temporal spacing between intervention, mediator, and outcome measurements will be included in the mediation analyses. The primary mediation model will be specified as a parallel path model in wide format. The path from treatment to the mediators will be modeled as follows:

$${M}_{1}\sim aX+ \gamma 1{M}_{0}$$

where *M*_1_ represents the mediator at posttreatment, $X$ represents the randomized treatment group (CBSST vs. control), and ${M}_{0}$ represents the baseline measure of the mediator. The indirect effect on the functioning outcome will be modeled as follows:

$${Y}_{2} \sim { c}{\prime}X+b{M}_{1}+\updelta {Y}_{0}+\updelta {M}_{0}$$

where ${Y}_{2}$ represents functioning at follow-up (6 or 12 months), ${Y}_{0}$ represents baseline functioning, and ${M}_{0}$ and ${M}_{1}$ are the baseline and posttreatment measures of the mediator, respectively. The indirect effect of treatment on functioning will be quantified as $a\times b$, the direct effect as ${c}^{{^{\prime}}}$, and the total effect as $c={c}^{{^{\prime}}}+a\times b$.

Additional exploratory models will test whether person-level covariates (e.g., baseline cognitive deficits, treatment exposure, age, or gender) or trial-level characteristics (e.g., delivery context) moderate any SEM paths through interaction terms with mean-centered predictors at level 1. Conditional indirect effects and their 95% confidence intervals will be reported for representative moderator values.

All models will be estimated using maximum likelihood with robust Huber–White standard errors to account for potential non-normality of residuals. For categorical indicators, weighted least squares estimation with mean and variance adjustment will be used. Indirect effects will be tested using bias-corrected bootstrapped confidence intervals based on cluster-respecting resampling at the participant level within trials. This approach provides robust estimates of uncertainty under non-normal sampling distributions of indirect effects. If bootstrap estimation proves computationally infeasible, confidence intervals will instead be derived using the delta method (i.e., Sobel or Monte Carlo approximation). Model fit will be evaluated using the chi-square test of model fit, the comparative fit index (CFI), Tucker–Lewis index (TLI), root mean square error of approximation (RMSEA), and standardized root mean square residual (SRMR). Prespecified thresholds (CFI/TLI ≥ 0.95, RMSEA ≤ 0.06, SRMR ≤ 0.08) will be used as general guidelines for acceptable fit, interpreted in conjunction with theoretical considerations, sample size, and residual diagnostics.

### Dose–response analysis

A dose–response meta-analysis of differences in means [54] adjusted to the one-stage IPD-MA framework will be used to examine the dose–response relationship between the cumulative number of CBSST sessions received and functioning at posttreatment and 6-month and 12-month follow-up, respectively. Dose will be modeled as a continuous exposure using a restricted cubic spline. This flexible, data-driven approach allows for the modeling of nonlinear dose–response relationships without imposing a specific functional form (e.g., linear or quadratic) and is recommended for dose–response meta-analyses [54]. Time will be treated as a categorical factor (i.e., posttreatment, 6 months, 12 months). The interaction term between time and the spline basis will be included as fixed effect to allow the dose–response to differ by follow-up. The model will be adjusted for baseline functioning in the fixed-effect term. Random effects will include a random intercept for trial, a random intercept for participant nested in trial, and a random time slope by study. The estimated model will yield a pooled, flexible dose–response curve with 95% confidence intervals, from which effective dose estimates (i.e., the number of sessions) associated with 50% and 80% of the maximum predicted improvement will be derived with 95% CIs via nonparametric bootstrap clustered by study at each assessment.

## Discussion

Motivational negative symptoms and the resulting functional impairment of people with schizophrenia represent highly important, yet unmet treatment targets. Cognitive Behavioral Social Skills Training is a promising evidence-based and targeted psychological intervention program that combines cognitive-behavioral techniques with social skills training to address the dysfunctional beliefs and social deficits common in schizophrenia. Despite many published clinical trials investigating the efficacy of CBSST on functioning and negative symptoms, the field currently lacks a meta-analytic aggregation of the overall effect sizes of CBSST on mental health outcomes as well as an empirically informed understanding of the relevant patient-level characteristics that might affect the effect of CBSST on the study outcomes. Insights into the overall efficacy of CBSST and relevant patient-level characteristics would improve our understanding of the benefits that can be expected from CBSST and could inform personalized treatment in clinical routine as well as the development of treatment guidelines for schizophrenia patients with negative symptoms and functional impairments.

To fill this gap and to better inform treatment planning, this individual-participant-data meta-analysis aims to provide a fine-grained synthesis of the evidence on the comparative efficacy of CBSST in patients with schizophrenia. Moreover, we will investigate patient-level (e.g., age, cognitive impairment) and study-level (e.g., additional digital intervention, group vs. individual format, active vs. passive comparator) predictors of treatment efficacy; explore the mechanisms of change, such as skill learning and cognitive attitudes, in facilitating improvements in functioning; and determine the dose of CBSST (number of sessions) needed to improve functioning.

This IPD meta-analysis is conceptualized to mitigate the limitations of previous meta-analyses that used classical aggregation approaches at the study level and, due to differing scopes, did not include the entire available evidence on CBSST. By leveraging the advantages of integrating more detailed information from IPD, we will be able to provide estimates with increased reliability and to explore factors that may influence these outcomes on the participant level. Thus, our study will significantly extend the existing literature on the treatment of functional impairment in patients with schizophrenia. 

## Limitations

Although an IPD meta-analysis can offer a more elaborate analysis necessary to provide more precise answers for this topic, it is more complex and time- and resource-intensive. A major challenge will be acquiring IPD, harmonization, and analysis of the IPD datasets. Our main goal is to harmonize these datasets into a common format, allowing a detailed synthesis of the evidence. It is anticipated that the majority of IPDs will originate from our own research group. While this facilitates high data availability and consistency in data structure, it also introduces potential biases, such as allegiance effects and restricted generalizability of findings. Notably, many IPD-MAs are not feasible due to the limited availability of IPD across studies. Therefore, the extensive access to IPD in the present project represents a major strength. Despite our direct access to most CBSST trial data, it may not be feasible to obtain IPD for all eligible studies or outcomes, particularly for those conducted by other research teams. To address potential data availability biases, we will systematically document reasons for missing IPD and apply meta-analytic models that allow the integration of studies providing IPD with those reporting only aggregate-level data. Because treatment dose is not randomized in the original studies and may be affected by early response, nonresponse, or dropout, estimates can only be interpreted as associations without causal inferences regarding the dose–response association in CBSST.

## Conclusion

It is pivotal to identify effective interventions for the management of severe functional impairment and negative symptoms in the treatment of patients with schizophrenia. Cognitive Behavioral Social Skills Training is a targeted psychological intervention program that has been demonstrated to alleviate negative symptoms and to enhance functional outcomes in a substantial number of clinical trials. Nevertheless, a comprehensive meta-analytic synthesis of the evidence, along with explicit evidence-based recommendations, remains to be undertaken. The findings of our individual-participant-data meta-analysis will provide the insights on the efficacy and differential indications to inform treatment decision-making and guideline recommendation development. Moreover, this study will identify potential gaps in the existing literature that may warrant further investigation. The findings will be published in a peer-reviewed scientific journal and subsequently disseminated in plain language through summaries and presentations to ensure broad accessibility to facilitate implementation of evidence-based interventions in clinical practice.

## Data Availability

The datasets analyzed during the current study are not publicly available due to data protection and confidentiality of individual participant data but are available from EG upon reasonable request.

## Abbreviations

CBSST: Cognitive Behavioral Social Skills Training
DSM IV and DSM 5: Diagnostic and Statistical Manual of Mental Disorders, Fourth and Fifth Editions
GRADE: Grading of Recommendations, Assessment, Development, and Evaluations
ICD-9 and ICD-10: International Classification of Diseases, Ninth and Tenth Editions
IPD: Individual participant data
IPD-MA: Individual participant data meta-analysis
PRISMA-IPD: Preferred Reporting Items for a Systematic review and Meta-Analysis of Individual Participant Data
RCTs: Randomized-control trials
RITES: Rating of Included Trials on the Efficacy-Effectiveness Spectrum tool
SCALE: Specify the problem, Consider all possible solutions, Assess the best solution, Lay out a plan, and Execute and evaluate the outcome
SMD: Standardized mean differences
ROB 2: Version 2 of the Cochrane risk-of-bias tool for randomized trials
